# Human sample﻿ authentication in biomedical research: comparison of two platforms

**DOI:** 10.1038/s41598-021-92978-3

**Published:** 2021-07-07

**Authors:** Harshitha Shobha Manjunath, Nicola James, Rebecca Mathew, Muna Al Hashmi, Lee Silcock, Ida Biunno, Pasquale De Blasio, Chidambaram Manickam, Sara Tomei

**Affiliations:** 1 Omics Core, Integrated Genomic Services, Research Branch, Sidra Medicine, PO 26999, Doha, Qatar; 2 Nonacus LtD, Birmingham, UK; 3 Integrated Systems Engineering, Milan, Italy

**Keywords:** Biological techniques, Molecular biology

## Abstract

Samples used in biomedical research are often collected over years, in some cases from subjects that may have died and thus cannot be retrieved in any way. The value of these samples is priceless. Sample misidentification or mix-up are unfortunately common problems in biomedical research and can eventually result in the publication of incorrect data. Here we have compared the Fluidigm SNPtrace and the Agena iPLEX Sample ID panels for the authentication of human genomic DNA samples. We have tested 14 pure samples and simulated their cross-contamination at different percentages (2%, 5%, 10%, 25% and 50%). For both panels, we report call rate, allele intensity/probability score, performance in distinguishing pure samples and contaminated samples at different percentages, and sex typing. We show that both panels are reliable and efficient methods for sample authentication and we highlight their advantages and disadvantages. We believe that the data provided here is useful for sample authentication especially in biorepositories and core facility settings.

## Introduction

Sample qualification is of paramount importance in biomedical research. Sample authentication is necessary to validate the data produced in any research project using human biosamples. Sample misidentification or mix-up are unfortunately common problems in biomedical research^1–5^ and can eventually lead to the publication of incorrect results^1,2,6,7^

Despite the cross-contamination of biological samples being a widely recognized problem^8^, only a minority of scientists perform tests to validate the identity of the samples analyzed prior to their studies^9,10^. Cell lines are considered “misidentified” when their genetic profile differs from that of the individual donors from whom they were initially established. Cell lines misidentification can be caused by the accidental substitution of culture samples or, perhaps most commonly, by cell line cross-contamination leading to the overgrowth of the contaminant cells resulting in cell mixtures^11^. Furthermore, during the growth of cell lines, genetic drift can occur due to their continued passage, making the identity testing not trivial to assess. It is estimated that between 10% and 35% of human cell lines are contaminated in some degree^12^.

In biobanks, sample authentication is generally required for reasons of different nature as compared to cell lines. In fact, biobanks store and process mostly samples of primary origin that do not undergo extensive in vitro manipulation and whose genomic footprint does not change during the experimental processing. Samples in these settings are most likely to be accidentally switched or mislabeled. Thus, sample authentication is of paramount importance to monitor all the steps the samples undergo to avoid the release of invalid data^13,14^.

There currently exist several methods for sample authentication, with the Single Tandem Repeat (STR) profiling being one of the methods initially employed^11,15^. Human STR profiling is based on a PCR reaction that targets polymorphic tetranucleotides or pentanucleotide repeats^16,17^. The differential number of repeats amplified produces DNA fragments of different sizes, which are given a numerical value after comparison to a set of size standards. The profile generated is characteristic of a given sample tested and can be entered into a database for comparison purposes^15,18^. The STR profiles made available by some cell banks generally include eight STR polymorphic loci plus amelogenin for gender determination^11,15^. Some other laboratories and cell biorepositories employ a larger number, typically 16 loci^19,20^. Recently, with the adoption of identity testing by laboratories, there is a need for systems that allow medium multiplexing (few tens of SNPs) with high throughput, that can generate efficient and cost-effective identity profiles on large sample sets. To address this need, several SNP panels have been employed for the detection of sample identity and relationship testing^21–26^, including the Agena iPLEX Sample ID and the Fluidigm SNPtrace panels.

The Agena iPLEX protocol encompasses a multiplex PCR, a multiplex single base extension (SBE) reaction and the detection of the SBE products by Matrix Assisted Laser Desorption/Ionization-Time of Flight Mass Spectrometry (MALDI-TOF MS). All the experimental reactions (PCR, Shrimp Alkaline Phosphatase (SAP) treatment, SBE and ion-exchange) are performed in the same plate until the samples are transferred to the SpectroCHIP for MALDI-TOF MS detection. Mass spectra are eventually analyzed and the SNPs called by the TYPER software on the MassARRAY analyzer system. Internal controls are also included for quality assurance in the PCR, SAP and SBE mixes^21^.

The Fluidigm SNPtrace testing is based on the use of an Integrated Fluidic Circuit (IFC), a network of fluid lines which provides precise metering of nanoliter fluid volumes and efficient mixing of the metered volumes. Once sample and assay mixes are dispensed into the integrated fluidic circuits, a PCR reaction is performed and the endpoint fluorescent image data is acquired using the BioMark HD System for genetic analysis. Data is analyzed using the Fluidigm SNP Genotyping Analysis software to obtain genotyping calls^27^.

Here we have tested the Agena iPLEX Sample ID Plus panel and the Fluidigm SNPtrace panel for authentication of human DNA samples. We have also simulated sample cross-contamination at different percentages to assess the efficiency of both panels in the detection of contamination.

## Methods

All methods were carried out in accordance with relevant guidelines and regulations.

### Samples

Fourteen DNA samples were employed in the study. Twelve samples were selected from the Congenital Heart Disease Biobank repository, one was a control DNA from CEPH individual (1347-02) and one sample was a laboratory control DNA isolated from a cardiomyocyte cell line, which we called LC1 as “Lab Control 1”. DNA was isolated from the blood samples and the cardiomyocyte cell line manually. The study was approved by the Sidra Medicine Institutional Review Board (IRB number:1500769-2). Informed consent was obtained from all subjects. The samples were randomly divided into 7 pairs. For each pair we used two reference samples (sample 1 and sample 2; Supplementary Table 1). DNA concentration was measured using the Quant-iT dsDNA Broad-Range Assay kit on the Qubit fluorometer (Thermo Fisher Scientific, Waltham, MA, USA). Samples were first normalized to the same concentration and then mixed to create cross-contamination at the following percentages: 2%, 5%, 10%, 25% and 50% for both sample 1 and sample 2 of each of the 7 sample pairs, thus the total number of samples tested is 84 (Supplementary Table 1). Samples were then genotyped using the Agena iPLEX Sample ID and Fluidigm SNPtrace panels. Positive and negative controls were used as appropriate for both panels.

### Agena iPLEX Pro sample ID

The Agena iPLEX Pro sample ID panel comprises 44 SNPs with high minor allele frequency across major HapMap populations, 3 gender markers and 5 control markers for DNA quality. The 44 SNPs and the 3 gender markers are used to generate the sample’s unique genetic fingerprint. Samples were genotyped using the iPLEX Pro sample ID panel and analysed using the MassARRAY system (Agena Bioscience, San Diego, USA). Briefly, a PCR was performed to amplify the target regions of interest followed by removal of unincorporated dNTPs using the Shrimp Alkaline Phosphatase (SAP); a Single Base Extension (SBE) was performed according to the manufacturer’s instructions with the supplied extension primers and the iPLEX reagents, as published elsewhere^21^. The sample plate was loaded onto the Agena Chip Prep Module CPM system which automatically desalts the extension product (analyte), approximately 15 nl of analyte and calibrant mix was transferred to the SpectroCHIP Array where it co-crystalized with the matrix and then it was transferred into the MassARRAY Analyser (a MALDI-TOF mass spectrometer) for the analysis. The genotyping data was acquired and processed by the SpectroTyper software. SNP calls were divided in three groups: “Conservative, Moderate and Aggressive genotype calls”. A fourth group, named “Low Probability SNP calls” contained genotypes with skewed allele balances or low signal-to-noise ratios. If no extended SBE primers were detected at any locus, the genotype call was labeled as “No allele”.

The MultiSampleID report software performed sample quality control, gender determination based on the three gender assays and sample identification using pairwise comparison of all sets of SNPs across samples.

### Fluidigm SNPtrace

Fluidigm genotyping was performed by parallel quantitative PCR (qPCR) using the high-throughput BioMark HD platform according to the manufacturer’s instructions. The genotyping assays were part of the SNPtrace panel. The Fluidigm SNPtrace panel Genotyping Assays (“SNPtrace panel”) is a set of SNPtype™ Assays for 96 single-nucleotide polymorphisms (SNPs) designed to provide information about the identity, gender, and quality of human genomic DNA samples. The panel includes 90 autosomal SNPs and 6 allosomal SNPs (3 Chr X SNPs and 3 Chr Y SNPs); the redundancy of the gender SNPs ensures that a sample can be gender-typed if one assay were to fail. The 90 autosomal SNPs include 46 loci highly polymorphic across all populations^28^ and 44 ancestry-informative loci^29^. The SNPtrace assay was run as per Fluidigm SNP genotyping user guide. Briefly, a specific target amplification (STA) was performed and the STA product was diluted 1:50 with DNA suspension buffer. Next, the assay primer mix was prepared by adding 3 μL of each allele-specific primer (ASP) and 8 μL of each locus-specific primer (LSP) for each of the 96 SNPtrace panel assays into a 96 well plate along with 29 μL of DNA suspension buffer. An assay mix was created by combining 1 μL of each assay primer mix and 2.5 μL of 2X Assay Loading Reagent and 1.5 μL of PCR-certified water. Next, the sample mix was prepared by combining 2.5 μL of the diluted STA product with 3 μL of Biotium 2X Fast Probe Master Mix, 0.3 μL of 20X SNPtype sample loading reagent, 0.1 μL of 60X SNPtype Reagent, 0.036 μL of ROX and 0.064 μL of PCR-certified water. Finally, 4 μL of assay mix were loaded into the assay inlets and 5 μL of each sample was loaded to the sample inlets of the Fluidigm 96.96 Dynamic Array integrated fluidic circuit (IFC) for genotyping. The BioMark HD dynamic array was first primed with control line fluid, and then loaded with the samples and assay mixtures via the appropriate inlets using the HX IFC Controller. The IFC array was placed in the BioMark HD for PCR using the following conditions: 95 °C for 10 min, 30 cycles at 95 °C for 15 s and 60 °C for 1 min according the protocol for genotyping plus Melt v2.pcl. The data was analyzed with Fluidigm SNP Genotyping analysis software (Fluidigm Corporation). Each PCR reaction used distilled water instead of DNA as negative control. Results were plotted on a two-dimensional scatter plot of the major versus the minor allele. Genotyping calls were assessed based on the allele discrimination plots and manually reviewed. The concordant calls between the pure and mixed samples were computed.

### Analysis

The 84 samples from the 7 randomly assigned pairs were given genotype calls for the 96 SNPs included in the Fluidigm SNPtrace panel and for the 47 SNPs included in the iPLEX Sample ID panel. The SNPs included in the Fluidigm SNPtrace and Agena iPLEX Sample ID Plus panels are listed in Supplementary Table 2. The genotype call of the pure samples for each pair was used as reference (Supplementary Table 1). Specifically, for the mixes in which sample 1 was “contaminated” with sample 2 at different ratios, we used sample 1 as reference. Vice versa, for the mixes in which sample 2 was “contaminated” with sample 1 at different ratios, we used sample 2 as reference (Supplementary Fig. 1).

A matrix including the genotype calls of the 84 samples assessed was generated for both panels. Concordance was defined based on the genotype similarity between the reference sample and the test sample. Specifically, the concordance between a reference sample and a test sample was calculated as the percentage of SNP assays with identical genotypes, where neither of the SNP assays gave a discrepant call; i.e. concordance = ((number of matching genotypes between test sample and reference sample)/(total number of SNPs)) * 100%. If both the reference and the test samples returned a no-call for a given SNP, that call was excluded from the calculation of concordance. The total number of SNP assays was 96 for the Fluidigm SNPtrace panel and 47 for the Agena iPLEX Sample ID Plus panel. The concordance values ranged between 0% (if no common genotypes were seen at any locus) and 100% (if the exact same genotypes were seen in both the samples at all loci). It should be noted that differently to other reports using the Tanabe algorithm to compute pairwise similarity^20,30^, we used genotype similarity to calculate concordance values (Supplementary Fig. 1)^31^. The concordance assessment in this study was based on the SNP composition, i.e. genotype match, rather than on the count of individual alleles. As an example, a homozygous A/A and a heterozygous A/B samples were considered discordant even if both samples shared the same A allele.

Pearson’s test was performed to assess the correlation between the Fluidigm SNPtrace and the Agena iPLEX Sample ID Plus concordance values. All statistical tests were based on two-tailed probability. A *p*-value < 0.05 was considered statistically significant. All the statistical analyses were performed using Statgraphics Centurion (V. 15, StatPoint, Inc.). Graphs were generated on Microsoft Excel for Mac, version 16.46, when relevant.

## Results

### Assessment of concordance for individual relatedness

Pairwise comparisons of the genotype calls were performed to assess the degree of similarity shared by the unrelated individuals. Mean and standard deviation of the pairwise concordance scores were computed for the “pure” samples (i.e. excluding the sample mixtures). The average concordance value for the Fluidigm SNPtrace panel across the unrelated samples was 46% (ranging from 26 to 72%, average ± st dev: 46.13 ± 8.64). The average concordance value for the Agena iPLEX Sample ID panel across the unrelated samples was 40% (ranging from 23 to 66%, average ± st dev: 40.143 ± 8.34).

We next sought to determine whether the two panels were consistent in the concordance value detection. By comparing the concordance values of the two platforms across the “pure” samples, we found a significant correlation (Pearson’s correlation R = 0.4063, 95% confidence interval: 0.2005 to 0.5778, *p*-value = 0.0002), suggesting a good consistency among the two platforms.

### Gender assignment

Sample gender was determined using the genotype calls of the gender SNPs. The Agena iPLEX Sample ID panel includes three gender markers, which utilize chromosome X and Y paralog sequences where the two paralogues are co-amplified by a single PCR primer pair and discriminated by differential bases or short INDEL at the single base extension level. Gender calls are returned as M = male, F = female and 0 = No Call. The gender assignment on the Fluidigm SNPtrace platform is based on six SNPs, three SNPs are located on the X chromosome and three SNPs on the Y chromosome. A sample was called “female” if the three X chromosome SNPs gave a call and the three Y chromosome SNPs gave a no-call. A sample was called “male” if the three Y chromosome SNPs and at least two of the three X chromosome SNPs gave a call. When both the X and Y chromosome SNPs gave a call with at least one of the X chromosome SNPs returning a heterozygous call, the sample was assigned as Klinefelter. In our dataset, contaminated samples with discordant gender (male/female) or male gender (male/male) returned a Klinefelter assignment.

Despite the number and the nature of the gender assays differing among the Fluidigm and Agena panels, the gender assignment was consistent across the two platforms for the “pure” samples assessed, except for the cardiomyocyte cell line LC1. The LC1 sample was assigned as male by the Fluidigm SNPtrace panel and as female by the Agena iPLEX Sample ID panel. To validate this finding we re-ran the LC1 for genotyping assessment. The sample was called as male by the Fluidigm SNPtrace panel and female by the Agena iPLEX panel also in the second run, consistently with the original gender assignment. Gender misassignment across different platforms has been previously reported in other studies^30,32–34^. Notably, in our study the only sample that had a discordant gender assignment by the two platforms is the immortalized cell line, while the DNA extracted from all the blood samples gave consistent gender assignment among the Fluidigm and Agena platforms. Immortalized cell lines might acquire aneuploidy and chromosomal rearrangements during the immortalization process. Since the gender assignment of the Fluidigm and Agena platforms is based on different assays, we believe that the discordance might be due to the genetic instability of the cell line assessed in this study.

### Detection of cross-contamination

**Figure 1:**
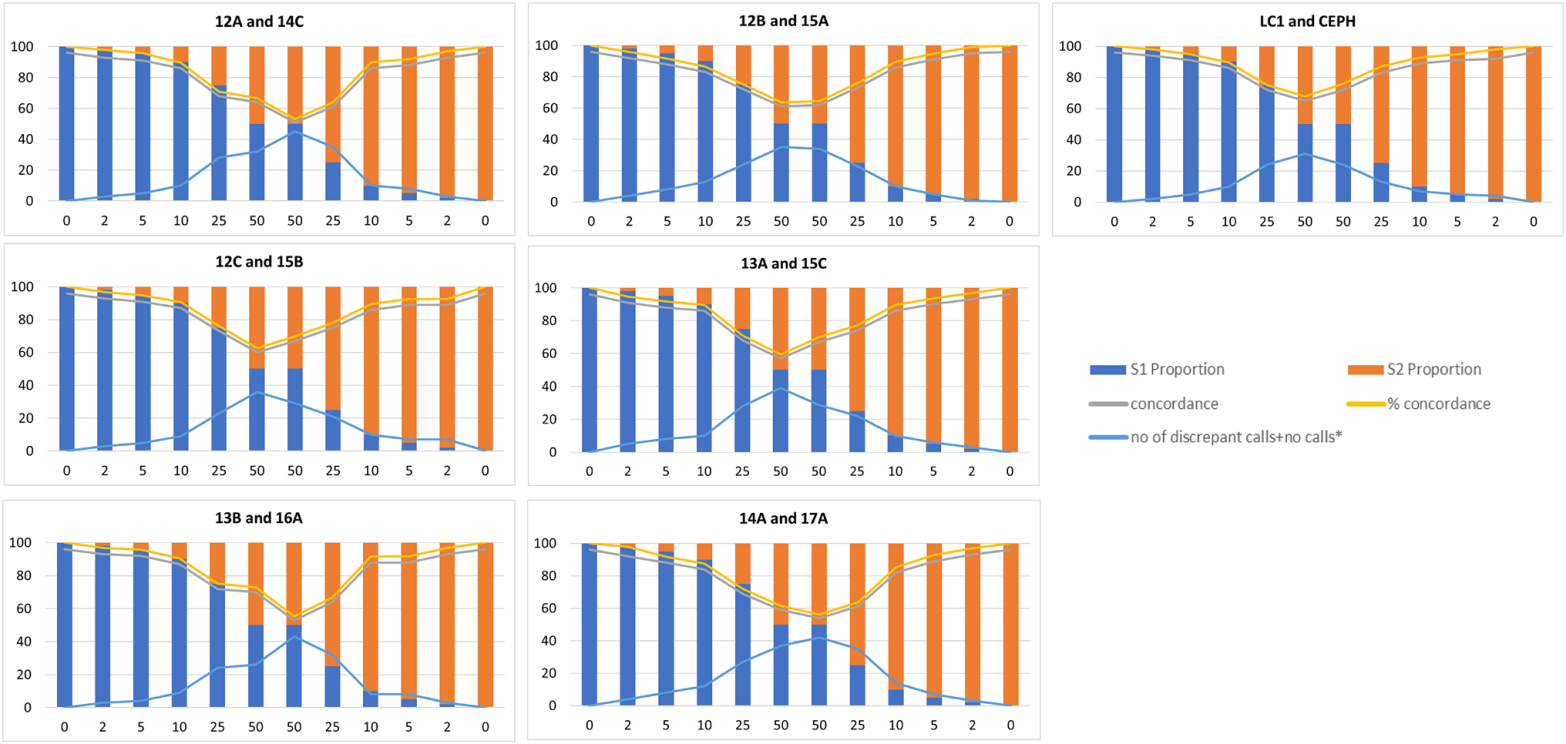
Concordance calls across the seven pairs of samples tested on the Fluidigm SNPtrace panel. The grey line indicates the concordant SNPs (N); the yellow line indicates the concordant SNPs (%); the blue line indicates the number of discordant plus no calls. Sample S1 is used as reference for the mixtures with S1 as the major component (blue) and the first 50:50 mixture; sample S2 is used as reference for the second 50:50 mixture and for the mixtures with S2 as the major component (orange).

**Figure 2:**
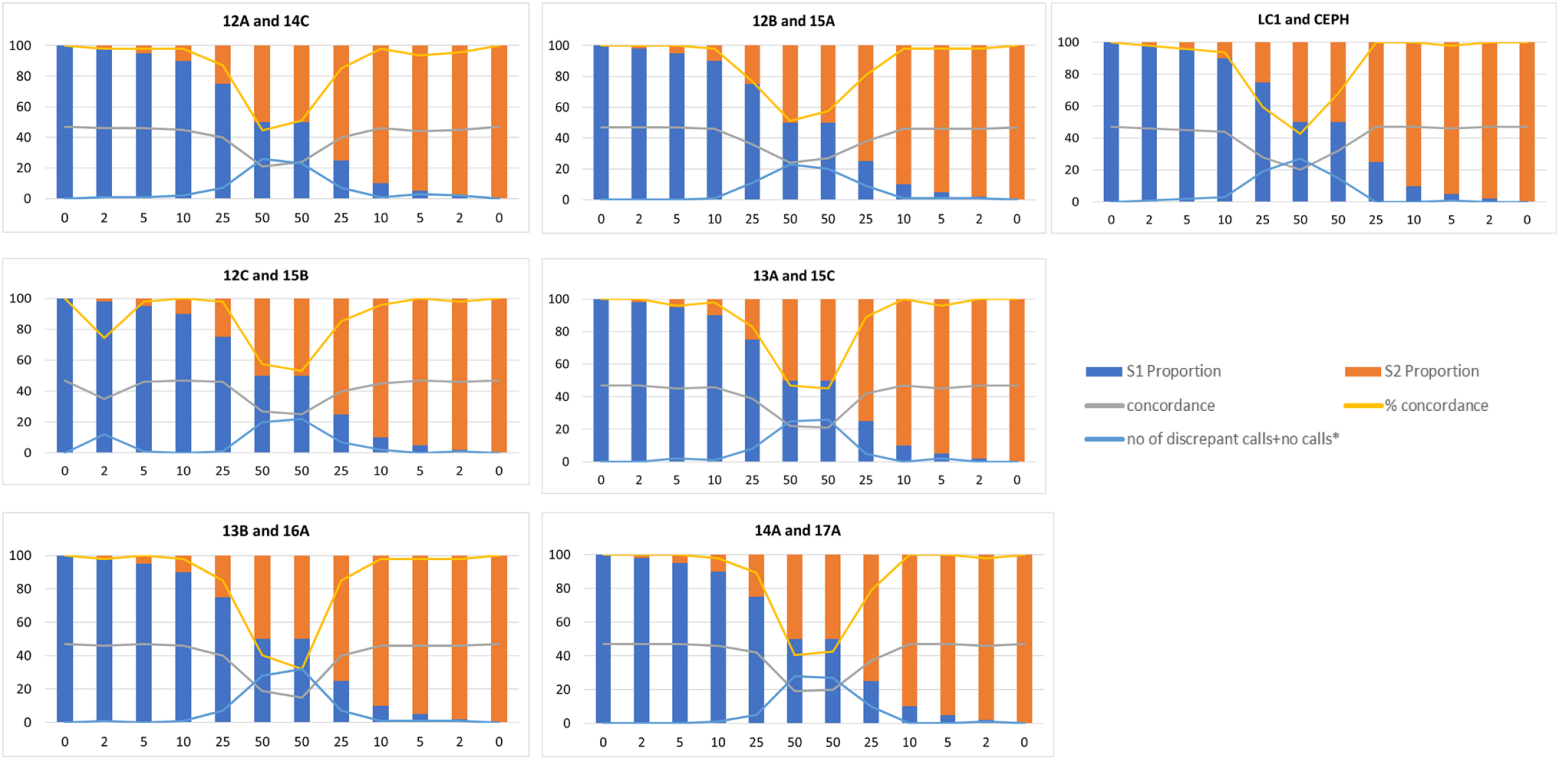
Concordance calls across the seven pairs of samples tested on the Agena iPLEX Sample ID Plus panel. The grey line indicates the concordant SNPs (N); the yellow line indicates the concordant SNPs (%); the blue line indicates the number of discordant plus no calls. Sample S1 is used as reference for the mixtures with S1 as the major component (blue) and the first 50:50 mixture; sample S2 is used as reference for the second 50:50 mixture and for the mixtures with S2 as the major component (orange).

**Figure 3:**
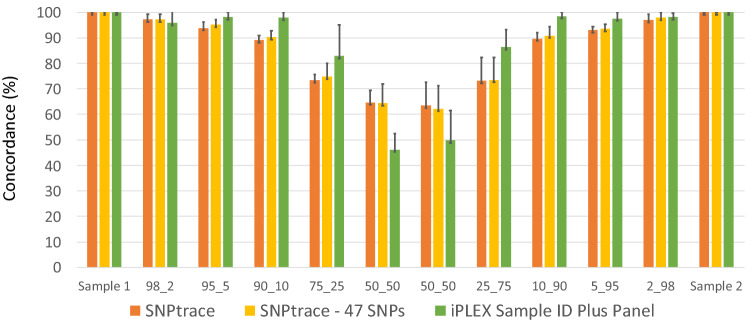
Average concordance (%) of the the SNPtrace panel (96 SNPs), SNPtrace panel (47 SNPs) and the iPLEX Sample ID Plus panel across the different sample mixes. Error bars indicate standard deviations.

To test the sensitivity of detecting cross-contamination, we created sample mixtures. The 14 individual samples were assigned to 7 random pairs; each pair of samples included “sample 1 (S1)” and “sample 2 (S2)”. Cross-contamination of the 7 pairs was simulated at fixed ratios of sample 1 and sample 2: 100:0; 98:2; 95:5; 90:10; 75:25; 50:50; 50:50; 25:75; 10:90; 5:95; 2:98; 0:100 (Supplementary Table 1). The 100:0 and 0:100 samples were considered “pure” samples. For the simulated contaminated samples, we used the genotype calls of the “pure” samples as reference (Supplementary Fig. 1). Concordance was calculated for the Fluidigm SNPtrace panel (Fig. 1) and the Agena iPLEX Sample ID panel (Fig. 2). It should be noted that for each sample pair we included two 50:50 experimental mixtures. This is because we used S1 as reference sample for the first mixture and S2 as reference sample for the second mixture. The number of discrepant calls + no calls increased parallelly to the increase of the contamination ratio (Figs. 1, 2) up to the first 50:50 mixture (using S1 as reference sample); from the second 50:50 mixture onwards the number of discrepant calls + no calls decreased parallelly to the decrease of the contamination ratio (using S2 as reference sample; Figs. 1, 2). The average concordance values of the Fluidigm SNPtrace and Agena iPLEX Sample ID panels across the cross-contaminated samples correlated significantly (Pearson’s correlation R = 0.8909, 95% confidence interval: 0.6488 to 0.9693, *p*-value = 0.0001). Nevertheless, the Fluidigm SNPtrace panel showed an overall greater drop in the concordance values with the parallel increase of the cross-contamination ratios as compared to the Agena iPLEX Sample ID panel (Fig. 3). Fluidigm SNPtrace allele discrimination plots for pair 12A/14C are shown as an example across all the contamination points in Supplementary Fig. 2. The tightness of the clusters reflects the call confidence. As expected, a greater dispersion is seen parallelly to increased contamination ratios, indicating a decreased discriminatory power when contamination increases.

Since the Agena iPLEX Sample ID Plus panel included a lower number of assays, we questioned whether a lower number of assays could decrease the discriminatory power of the Fluidigm SNPtrace panel. To test this hypothesis, we randomly selected 47 SNPs and assessed the concordance values. Concordance was evaluated based on the number of SNPs (N) and the percentage of SNPs that gave a concordant call over the total discriminative SNPs (Supplementary Fig. 3). The mean concordance value for unrelated samples changed negligibly when the SNP count was reduced from 96 to 47, while the standard deviation increased slightly (Fig. 3). Overall, for most of the mixtures the Fluidigm SNPtrace panel showed a greater drop of the concordance values even when randomly selecting 47 SNPs, suggesting a greater ability of the Fluidigm SNPtrace panel to discriminate contamination, whereas the Agena iPLEX Sample ID Plus panel showed a better discriminatory power for the the 50:50 mixtures (Fig. 3).

**Figure 4:**
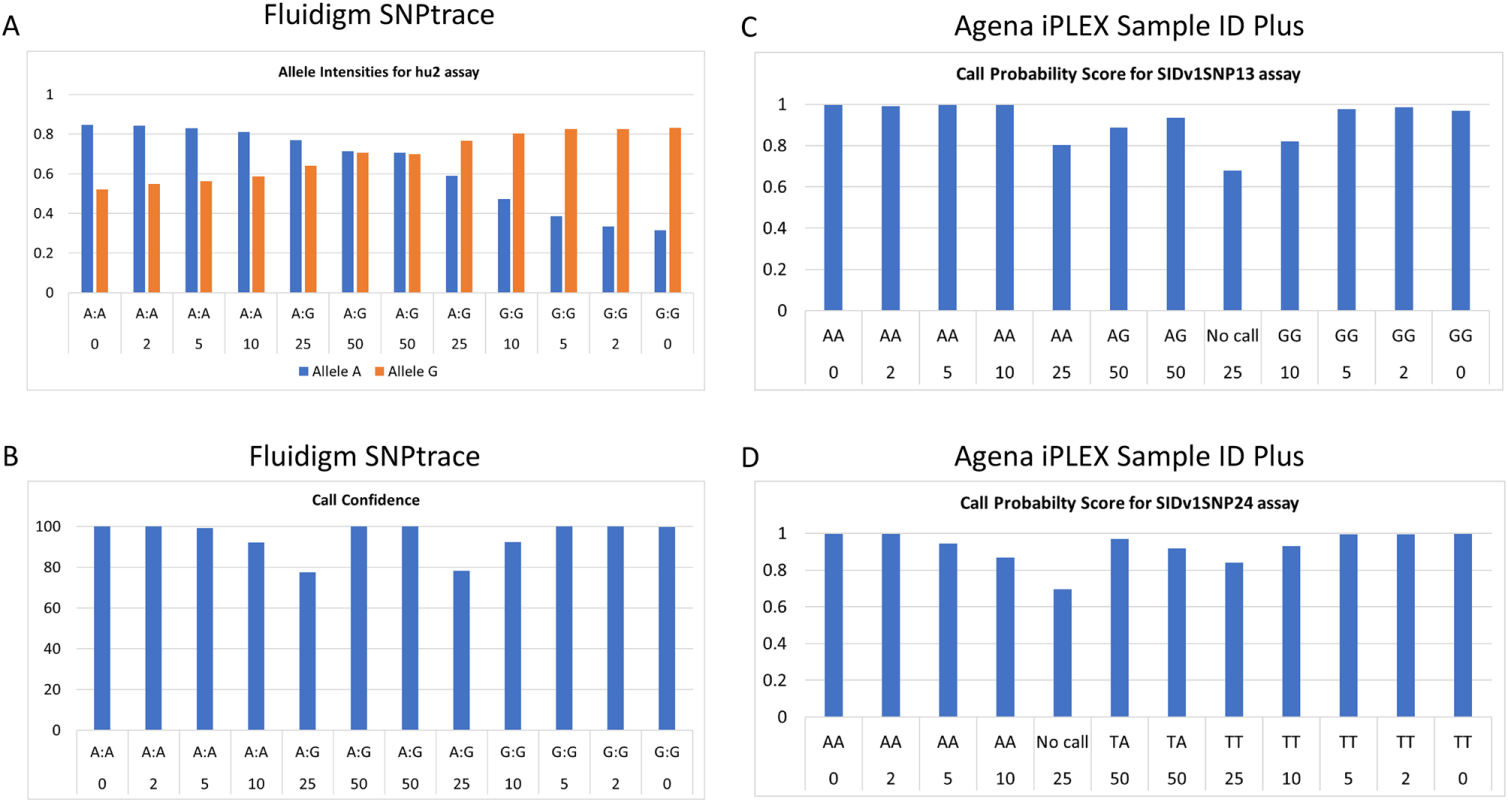
Allele intensities (**A**) and Call Confidence (**B**) plots for Fluidigm SNPtrace hu2 assay for sample pair 12A and 14C. Call Probability Score for Agena iPLEX Sample ID Plus SNP13 assay (**C**) and SNP24 assay (**D**).

We next looked at the allele intensity/call confidence scores and the call probability scores of the Fluidigm SNPtrace and the Agena iPLEX Sample ID Plus panel, respectively, as a measure of the call reliability. We used hu2 assay as an example for the Fluidigm SNPtrace panel and looked at the allele intensity and call confidence for the 12A (homozygous A/A) and 14C (homozygous G/G) sample pair (Fig. 4A,B). The G and A allele intensities were > 0.3 for the A/A and G/G “pure” samples respectively, indicating a significant elevated level of fluorescence for both alleles. As expected, the intensity of the “contaminant” alleles increased progressively with the increased contamination ratio, while the call confidence decreased parallelly; however, the call confidence increased when the contamination ratio was 50:50 as both alleles are represented equally. A similar trend was noticed for the call probability score of the Agena iPLEX Sample ID Plus assays, examples of SNP13 and SNP24 assays are reported in Fig. 4C,D. Thus, in the case of two samples homozygous for two different alleles of a given SNP, the assessment of the call confidence for the Fluidigm SNPtrace panel and of the call probability score for the Agena iPLEX Sample ID Plus panel would help in identifying sample mixtures where the contaminant represents at least 10% of the sample, whereas contaminants representing 2% and 5% of the samples would be the most difficult to discern.

### Experimental processing comparison

We have evaluated the processing time and easiness of the Fluidigm SNPtrace panel (Table 1) and Agena iPLEX Sample ID Plus panel (Table 2). The entire protocol for the Fluidigm SNPtrace panel takes around 7 h. The protocol is relatively easy to follow. The transfer of samples and primers must be done carefully.

**Table 1:** Experimental steps for Fluidigm SNPtrace panel.

Experimental steps	Hands-on time	Run time
Specific target amplification (STA)	30 min	1 h 15 min
SNP Trace panel Assay and sample preparation	1 h	–
IFC priming	–	20 min
Sample and assay transfer to IFC and loading	15 min	1 h 30 min
Thermal cycling and reading on Biomark HD	–	1 h 30 min
QC analysis	10 min	–
Total	1 h 55 min	4 h 35 min

**Table 2:** Experimental steps for Agena iPLEX Sample ID Plus panel.

Experimental steps	Hands-on time	Run time
PCR	10 min	2.5 h
SAP	5 min	1 h
Extension	10 min	3.5 h
Target sequence detection	5 min	1 h 30 min
Data analysis	5 min	–
QC report	5 min	
Total	40 min	8 h 30 min

The iPLEX Pro sample ID panel workflow is a single day turnaround consisting in three major steps, namely the PCR/SAP/Extension, Target Sequence Detection and the Data QC and reporting. The workflow is relatively easy to follow. A comparison of the two platforms is provided in Tables 1 and 2. Although the processing time for the Agena iPLEX Sample ID Plus panel is a little longer than the Fluidigm SNPtrace panel, the experimental steps of both platform are comparable.

Notably, the Agena iPLEX Sample ID Plus panel requires ~ 6 times less DNA compared to the Fluidigm SNPtrace panel, this is an important consideration to take into account when working with limited DNA amount.

## Discussion

DNA-based authentication is of critical importance to validate the results of any research. It is unfortunate that researchers have only recently begun dedicating more attention to the possibility of sample misidentification and its associated problems^8,13,14^. Tracking samples from acquisition to data analysis is particularly important for biobanks and centralized core facilities. Sample mix-ups and contaminations are common problems in molecular laboratories; identifying these instances helps biobanks and core facilities to maintain high reliability and credibility. A standard genotyping workflow should be implemented to confirm the identity of each sample and assess their quality prior to analysis. Sample handling errors can occur before and/or after the samples enter the core laboratory or the storage facility and when they are transferred among collaborating researchers. A specific genotyping workflow should be developed for authentication. In fact, when samples are processed for molecular analyses they undergo several steps; each of these steps is prone to accidental handling errors that may compromise the samples and invalidate related data. In biobanks, sample authentication processes are necessary to ensure that specific samples came from a given donor before they get distributed to the scientific community. In an ideal setting, an internal SNP database should be established and should include SNP data of all the samples collected, stored and distributed by the biobank. At best, SNP profiling should be performed at the time of sample collection and prior to sample distribution; this is to ensure consistency and to identify samples that might have been accidentally mislabeled or switched.

Especially when planning large scale studies and collecting large volumes of samples it is important to maintain samples traceability^14^. Sample traceability can be achieved by DNA fingerprinting, which is of crucial importance in biobanking and core facility settings^13^. Several DNA fingerprinting methods currently exist. However, when applied to biobank and core facility settings they need to be rapid, reliable, low-cost and simple^13,35^. In this study, we have tested the Fluidigm and Agena platforms for sample authentication. We have evaluated the discriminatory power to detect contamination at different ratio.

We found that the Fluidigm SNPtrace panel performed slightly better than the Agena iPLEX Sample ID Plus panel in detecting contamination at lower ratios. Specifically, the Fluidigm SNPtrace panel had a better discriminatory power for all the sample mixtures except the 50:50 mixtures, which were better detected by the Agena iPLEX Sample ID Plus panel. As the Fluidigm SNPtrace panel includes a greater number of assays compared to the Agena iPLEX Sample ID Plus panel, we questioned whether selecting a similar number of assays from Fluidigm SNPtrace panel would have resulted in a similar performance compared to the Agena iPLEX Sample ID Plus panel. As expected, reduction of the number of loci detected resulted in a slight decrease of the discriminating ability, however the Fluidigm platform resulted in a better discriminatory power as compared to the Agena platform even when randomly selecting 47 assays across the cross-contamination pairs. Nevertheless, the Agena iPLEX Sample ID Plus discriminatory power was greater when the cross-contamination ratio was 50:50. The overall experimental time was a little longer for the Agena iPLEX Sample ID Plus panel as compared to the Fluidigm SNPtrace panel, although the Agena workflow required less hands-on time as compared to the Fluidigm workflow. Agena required a lower DNA input amount which is an important point to consider when having only limited samples.

The gender assignment was also consistent across the two platforms except for the cell line sample. Immortalized cell lines are known to have a greater degree of aneuploidy and chromosomal rearrangements due to the genomic instability acquired during the immortalization process. The genomic instability might affect gender chromosomes. Nevertheless, the gender assignment was correct and consistent across the two platforms for all the DNA samples isolated from blood.

Overall, Agena and Fluidigm platforms performed well in detecting cross-contamination. Similar to others, we show here that SNP-based profiling is able to detect intra-human cross contamination as low as 2%^30,36^, which seemed to be favorable as compared to STR profiling^30^. In our experience, the Fluidigm SNPtrace panel has allowed the resolution of three cases including a mixed cell line and two switched samples cases. This should support its implementation in biobank and core facility settings.

## Conclusion

In conclusion, we show here that both the SNPtrace and the iPLEX Sample ID Plus panels are valid tests for sample authentication; consistency in genotyping assignment was found across the two platforms. The SNPtrace panel showed good performance in the detection of contamination. Although the iPLEX Sample ID Plus panel showed a relatively lower sensitivity as compared to the Fluidigm SNPtrace panel, the panel may be useful for identification of samples in human biobanks because of the limited DNA amount required for the test, as in the case of biopsies and formalin-fixed paraffin-embedded tissues. It should also be noted that the results reported here are based on good-quality DNA samples. Additional tests are warranted to understand whether similar results can be obtained when using DNA samples of lower quality (e.g. DNA isolated from formalin-fixed paraffin-embedded tissues).

## Supplementary Information

Supplementary Figures.

Supplementary Table 1.

Supplementary Table 2.

Supplementary Legend.

## Abbreviations

ASP: Allele specific primer
CPM: Chip prep module
IFC: Integrated fluidic circuit
LSP: Locus specific primer
MALDI-TOF-MS: Matrix assisted laser desorption/ionization-time of flight mass spectrometry
PCR: Polymerase chain reaction
SAP: Shrimp alkaline phosphatase
SD: Standard deviation
SBE: Single base extension
SNP: Single nucleotide polymorphism
STA: Specific target amplification
STR: Single tandem repeat
