# Supplementary material for: Human sample﻿ authentication in biomedical research: comparison of two platforms
Source: Sci Rep. 2021 Jul 7;11:13982. doi: 10.1038/s41598-021-92978-3 (PMC8263568; doi:10.1038/s41598-021-92978-3)

Human sample authentication in biomedical research: comparison of two platforms

Harshitha Shobha Manjunath[1], Nicola James[2], Rebecca Mathew[1], Muna Al Hashmi[1], Lee Silcock[2], Ida Biunno[3], Pasquale De Blasio[3], Chidambaram Manickam[1], Sara Tomei[1,*]

[1]Omics Core, Integrated Genomic Services, Research Branch, Sidra Medicine, Doha, Qatar

[2]Nonacus LtD, Birmingham, UK

[3]Integrated Systems Engineering - Milan, Italy

Supplementary Figure 1

A Example of sample mixes

Sample ID	S1 (%)	S2 (%)	Reference
Sample 1 (S1)	100	0	S1
S1(98):S2(2)	98	2	S1
S1(95):S2(5)	95	5	S1
S1(90):S2(10)	90	10	S1
S1(75):S2(25)	75	25	S1
S1(50):S2(50)	50	50	S1
S1(50):S2(50)	50	50	S2
S1(25):S2(75)	25	75	S2
S1(10):S2(90)	10	90	S2
S1(5):S2(95)	5	95	S2
S1(2):S2(98)	2	98	S2
Sample 2 (S2)	0	100	S2

B Example of matrix reporting concordance values of a sample pair (%, SNPtrace)

	Sample 1 (S1)	S1(98):S2(2)	S1(95):S2(5)	S1(90):S2(10)	S1(75):S2(25)	S1(50):S2(50)	S1(50):S2(50)	S1(25):S2(75)	S1(10):S2(90)	S1(5):S2(95)	S1(2):S2(98)	Sample 2 (S2)
Sample 1 (S1)	100	98	96	90	71	67	66	48	40	40	40	40
S1(98):S2(2)	98	100	97	91	71	67	66	48	40	40	40	40
S1(95):S2(5)	96	97	100	93	73	69	68	50	40	40	40	40
S1(90):S2(10)	90	91	93	100	77	73	72	53	40	40	40	40
S1(75):S2(25)	71	71	73	77	100	84	83	66	42	42	42	41
S1(50):S2(50)	67	67	69	73	84	100	99	78	54	54	54	53
S1(50):S2(50)	66	66	68	72	83	99	100	78	54	54	54	53
S1(25):S2(75)	48	48	50	53	66	78	78	100	71	70	66	64
S1(10):S2(90)	40	40	40	40	42	54	54	71	100	97	93	90
S1(5):S2(95)	40	40	40	40	42	54	54	70	97	100	95	92
S1(2):S2(98)	40	40	40	40	42	54	54	66	93	95	100	97
Sample 2 (S2)	40	40	40	40	41	53	53	64	90	92	97	100

C

$$\text{Concordance} = \frac{\text{(N matching genotypes)}}{\text{(total SNPs)}} \times 100$$

$$\text{Concordance SNPtrace} = \frac{48}{96} \times 100 = 50\%$$

Supplementary Figure 2

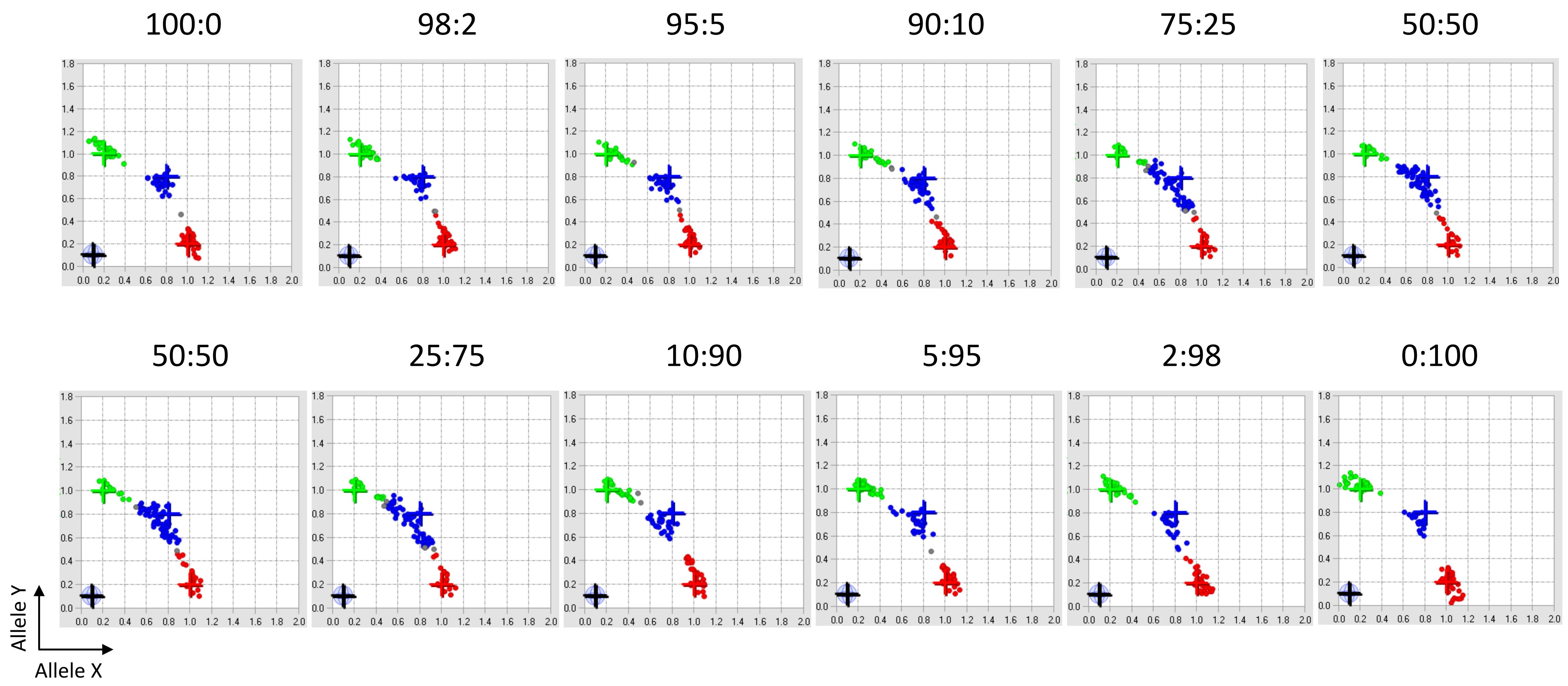

Supplementary Figure 3

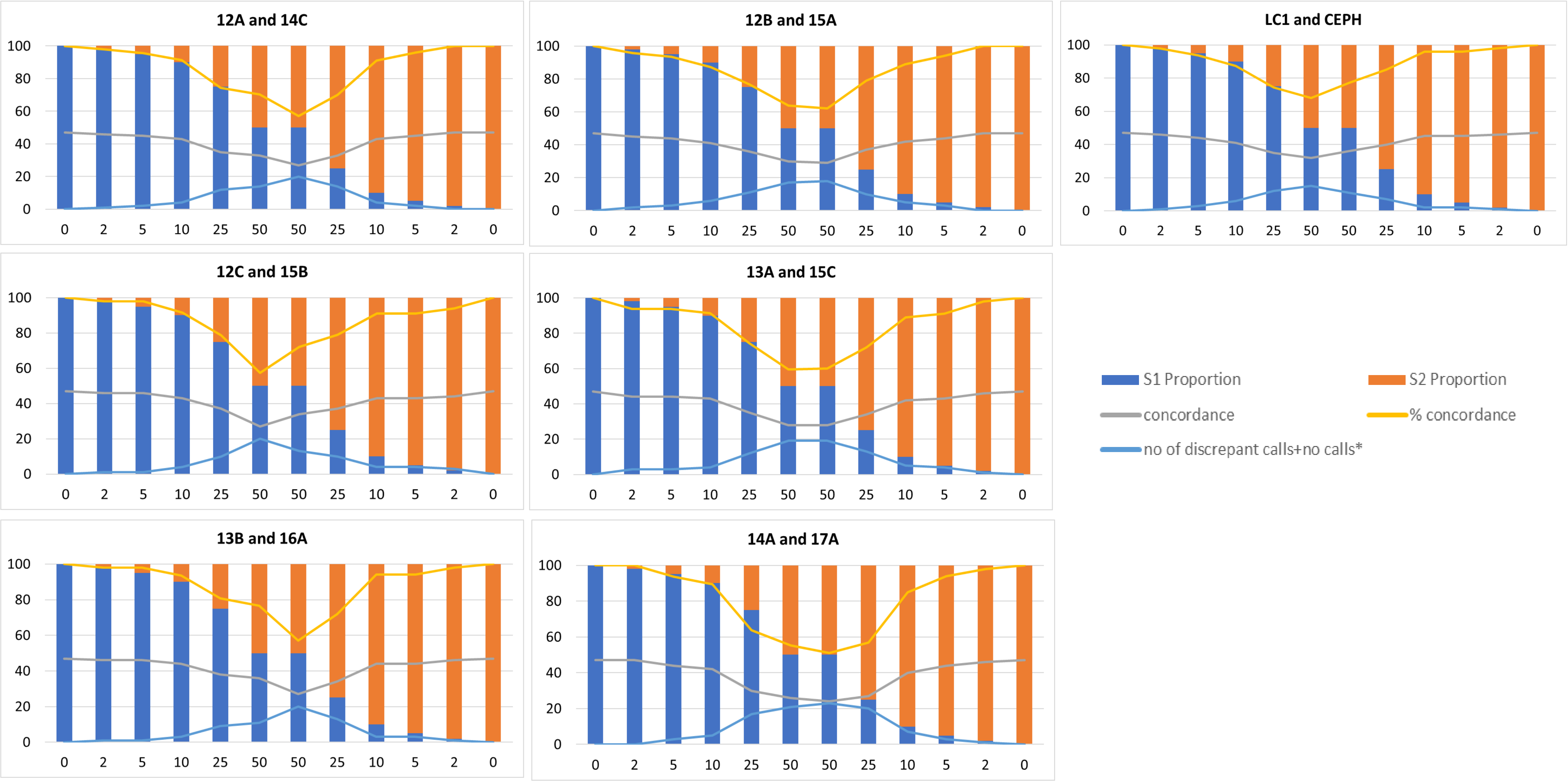

Supplement: Supplementary file 1 — Supplementary Figures. [file 41598_2021_92978_MOESM1_ESM.pdf]
