# Supplementary material for: Human sample﻿ authentication in biomedical research: comparison of two platforms
Source: Sci Rep. 2021 Jul 7;11:13982. doi: 10.1038/s41598-021-92978-3 (PMC8263568; doi:10.1038/s41598-021-92978-3)
Supplement: Supplementary file 4 — Supplementary Legend. [file 41598_2021_92978_MOESM4_ESM.docx]

**Supplementary Material**

**Supplementary Table 1.** List of the 84 samples included in the study. The table reports the cross-contamination ratios of the 7 sample pairs along with the concordance values for the autosomal and gender assays for both the Fluidigm SNPtrace and Agena iPLEX Sample ID Plus panels.

**Supplementary Table 2**. List of assays included in the Fluidigm SNPtrace and the Agena iPLEX Sample ID Plus panel.

**Supplementary Figure 1**. Example of sample mixtures for each of the seven pairs assessed in our study (A). Example of concordance matrix for a sample pair (B). Formula used for the calculation of the concordance values (C).

**Supplementary Figure 2**. Allele discrimination plots of all the SNPs included in the Fluidigm SNPtrace panel for sample pair 12A and 14C. Each dot represents a SNP. For each sample contamination ratio, homozygous allele 1, heterozygous and homozygous allele 2 calls are represented in green, blue and red, respectively. No calls are in grey.

**Supplementary Figure 3**. Concordance calls across the seven pairs of samples tested on the Fluidigm SNPtrace panel using 47 randomly selected SNPs. The grey line indicates the concordant SNPs (N); the yellow line indicates the concordant SNPs (%); the blue line indicates the number of discordant plus no calls. Sample S1 is used as reference for the mixtures with S1 as the major component (blue) and the first 50:50 mixture; sample S2 is used as reference for the second 50:50 mixture and for the mixtures with S2 as the major component (orange).
